# Dinutuximab beta in the treatment of high-risk neuroblastoma: A follow-up of a case series in Bratislava

**DOI:** 10.1097/MD.0000000000028716

**Published:** 2022-01-28

**Authors:** Monika Achbergerová, Stanislava Hederová, Andrea Hrašková, Alexandra Kolenová

**Affiliations:** Department of Paediatric Haematology and Oncology, National Institute of Children's Diseases Bratislava, Slovakia.

**Keywords:** anti-GD2 therapy, case series, dinutuximab beta, high-risk neuroblastoma, single-center experience

## Abstract

Despite therapeutic advances, high-risk neuroblastoma is still associated with a poor long-term prognosis. Immunotherapy with the anti-GD2 antibody dinutuximab beta has recently been added to the standard of care for patients with high-risk neuroblastoma in our center in Bratislava, and our initial experience with dinutuximab beta has been reported previously. Here we provide a follow-up on the outcomes of 7 patients who were treated with dinutuximab beta under clinical practice conditions at our center.

Medical records of 31 patients diagnosed with neuroblastoma between 2017 and 2020 at the Children's Hematology and Oncology Clinic in Bratislava were retrospectively reviewed and 7 patients with high-risk neuroblastoma who were treated with dinutuximab beta were identified. All 7 patients received dinutuximab beta as continuous infusion over 10 days at a dose of 10 mg/m^2^/day for 5 cycles, following induction and consolidation therapy. Supportive therapy was administered to manage adverse events. Clinical outcomes and treatment tolerance were evaluated.

Six of 7 patients treated with dinutuximab beta achieved complete remission, with a median duration of response of 21.5 months as of January 2022, and 1 displayed stable disease 21 months after treatment completion. Treatment was tolerable in most patients, with the majority of adverse events managed with supportive care.

Dinutuximab beta is an effective immunotherapy for patients with high-risk neuroblastoma in routine clinical practice when coupled with optimal supportive management of adverse events.

## 1 Introduction

Neuroblastoma is an embryonal tumor originating in immature cells of the sympathetic nervous system. It is the most common extracranial solid tumor in children, representing 7% to 10% of all childhood malignancies and accounting for 15% of deaths in pediatric oncology.^[1]^ Neuroblastoma has an extremely heterogenous clinical course, with some tumors regressing completely, while others progress and metastasize. Treatment is based on pretreatment risk stratification according to the International Neuroblastoma Risk Group (INRG) staging system, which takes into account disease stage, patient's age at diagnosis, tumor histology and genetic profile (including *MYCN* status, segmental chromosomal aberrations and DNA ploidy).^[2]^ Intensity of treatment and prognosis depend on this assessment, with therapeutic strategies for patients with high-risk neuroblastoma being amongst the most aggressive and longest lasting regimens in pediatric oncology. In recent years, harnessing the body's own immune system via immunotherapies has proven to be an effective strategy in many cancer types,^[3]^ and addition of immunotherapy agents to existing cytotoxic regimens has demonstrated improved outcomes for patients with neuroblastoma.^[3–6]^

The glycosphingolipid disialoganglioside (GD2) is an established tumor-associated antigen present on multiple tumor types, including neuroblastoma, with restricted expression in normal tissues.^[5,7]^ Dinutuximab beta is an anti-GD2 monoclonal antibody, which binds to GD2 on tumor cells, marking these cells for destruction by the body's own immune system.^[5,7]^ Dinutuximab beta has improved survival rates in neuroblastoma and has been approved in Europe for use in high-risk patients, based on a Phase III trial lead by the SIOPEN (International Society of Paediatric Oncology Europe Neuroblastoma) group.^[4,8,9]^ It has now become the standard of care as part of maintenance therapy for patients with high-risk neuroblastoma who have achieved at least a partial response to previous multimodal treatment.^[7,9,10]^ However, published data on the use of dinutuximab beta in routine clinical practice is still sparse.

We previously reported our initial experience with the introduction of dinutuximab beta in our clinic in Bratislava.^[11]^ The aim of this article is to provide more details on the 7 patients with newly diagnosed, localized or metastatic high-risk neuroblastoma treated under real-world conditions and report on their longer term clinical outcomes and treatment tolerance.

## 2 Materials and methods

Medical and nursing records of 31 patients diagnosed with neuroblastoma between 2017 and 2020 at the Children's Hematology and Oncology Clinic in Bratislava were retrospectively reviewed to identify patients with high-risk neuroblastoma treated with dinutuximab beta (EUSA Pharma BV, Netherlands) and evaluate their treatment responses and adverse events (AEs). Informed consent for study participation was obtained from the patients’ parents.

Seven patients were stratified as high-risk and treated according to the SIOPEN protocol. All 7 patients had completed multimodal therapy before beginning dinutuximab beta treatment: induction chemotherapy with rapid cisplatin, carboplatin, cyclophosphamide, vincristine, etoposide, surgery, myeloablative chemotherapy with BuMel (busulfan/melphalan) followed by autologous stem cell transplantation (ASCT), and radiotherapy focused on the primary tumor site and other active sites. Dinutuximab beta was administered as maintenance therapy as continuous intravenous infusion over 10 days at a dose of 10 mg/m^2^/day, with each patient receiving five 28-day cycles. Differentiation therapy with 13-cis-retinoic acid 160 mg/m^2^/day was also administered over 14 days (6 cycles in total). Supportive therapy was provided, which consisted of intravenous hydration, combined prophylactic analgesia (opioids and non-opioids, prophylaxis of neuropathic pain), antiallergic and antiemetic prophylaxis. Patients were intensively monitored, and supportive therapy was de-escalated in a step-wise fashion if no complications were observed. Corticosteroids, immunoglobulins, and other immunosuppressive and immunomodulatory therapies were contraindicated 2 weeks before, during, and 2 weeks after completing dinutuximab beta therapy.

Outcomes of interest were clinical response and treatment tolerability. Tumor progression and metastatic status were evaluated using computed tomography, magnetic resonance imaging, meta-iodobenzylguanidine (MIBG), and/or ultrasound scans after induction therapy, after ASCT, after radiotherapy, and after the last cycle of dinutuximab beta treatment. AEs were assessed using the National Cancer Institute's Common Terminology Criteria for Adverse Events (version 4.03 ).

## 3 Results

Dinutuximab beta was implemented in the treatment of high-risk neuroblastoma at our department in June 2018. As of January 2022, 7 patients with high-risk neuroblastoma completed treatment with dinutuximab beta. The patient and disease characteristics are presented in Table 1. The median age at diagnosis was 22.5 months (range 13.1–52.8 months); 3 patients were male and 4 were female. All patients presented with poorly differentiated neuroblastoma with segmental chromosomal aberrations; 4 patients also had tumors with confirmed *MYCN* amplification. At the time of diagnosis, 6 patients were classified as metastatic disease (stage M) and one patient had localized disease (stage L2).

**Table 1:** Key details of the patients’ diagnosis, characteristics of the tumor, and treatment outcome.

	Patient 1	Patient 2	Patient 3	Patient 4	Patient 5	Patient 6	Patient 7
Age at diagnosis (months)	22.5	18.9	13.1	38.3	52.8	21.4	32.5
Sex	Male	Male	Female	Female	Female	Male	Female
*MYCN* status	+	−	+	−	−	+	+
SCA status	+	+	+	+	+	+	+
INRG stage	L2	M	M	M	M	M	M
Signs and symptoms	Irritability lasting over two weeks, extensive sweating and abdominal pain	Mild left eye protrusion	Progressive left eye protrusion and periorbital hematoma	Hip pain, lump behind left ear	Hip pain, coxitis and elevated inflammatory markers	Severe anemia and elevated inflammatory markers	Abdominal pain and constipation
Site of primary tumor	Left retroperitoneum	Left retroperitoneum	Right retroperitoneum	Right lung apex	Left posterior mediastinum	Left retroperitoneum	Left retroperitoneum
Site of metastases	None	Regional lymph nodes, bone marrow, skeleton (skull with soft tissue component, scapula, ribs, pelvis, femur bilateral)	Skeleton (splanchno/neurocranium, sternum, scapula, ribs, humerus, pelvis, limb bones), bone marrow, liver, retroperitoneal lymph nodes, conus medullaris, lumbar nerve roots, intraspinal extradural tumor	Retroperitoneal lymph nodes, bone marrow, skeleton (occipital bone with soft tissue component)	Lymph nodes, bone marrow	Retroperitoneal lymph nodes, skeleton (vertebrae, illium), intraspinal extradural tumor	Bone marrow
Response prior to dinutuximab beta	CR	VGPR	VGPR	CR	VGPR	CR	CR
Dinutuximab beta cycles	5	5	5	1^∗^	5	5	5
Response at last follow-up	CR	VGPR	CR	CR	CR	CR	CR
Duration of response (months)^†^	27	18	16	24	21	12	6

∗ Patient discontinued after the first cycle due to severe neurotoxicity.

† As of October 2021. CR = complete response, INRG = International Neuroblastoma Risk Group, L2 = tumor has not spread beyond the area where it started and the nearby tissue, M = tumor has spread to other parts of the body, *MYCN* = proto-oncogene protein, SCA = segmental chromosomal aberration, VGPR = very good partial response.

Two patients presented with abdominal pain, 2 with hip pain, and 2 exhibited left eye protrusion. In 5 patients, the primary tumors occurred in the retroperitoneum, whereas the other 2 patients displayed tumors in the posterior mediastinum and the apex of the lung. Patients 2 and 3 displayed additional tumorous lumps in the left temporal region causing the eye protrusion; patient 4 had a tumor in the retroauricular region. Bone marrow infiltration and/or lymph node metastases were observed in all patients with metastatic disease; some patients also exhibited metastases in other sites, such as the skeleton, kidney, liver or spinal cord.

**Figure 1:**
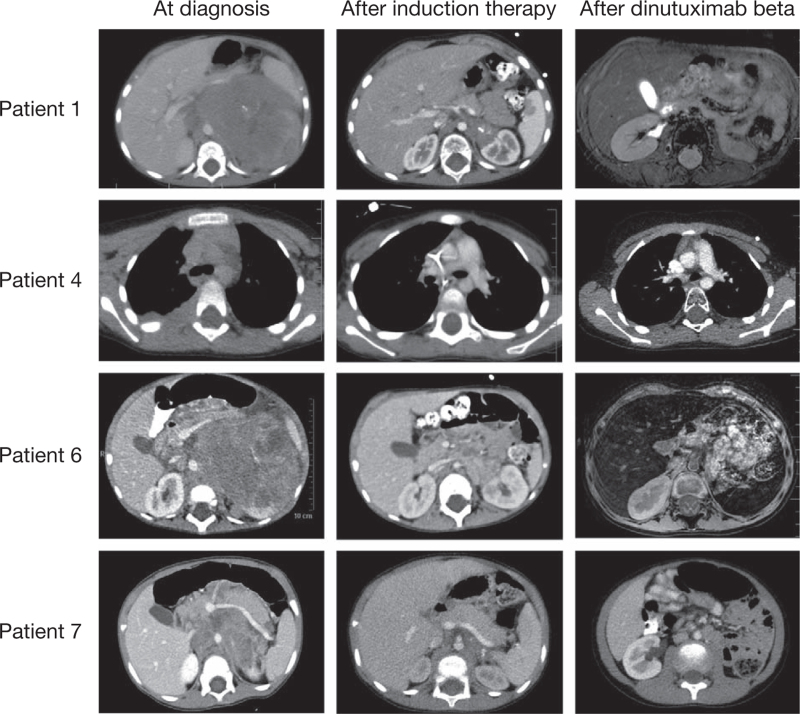
Abdominal/thorax CT or MRI scans at diagnosis (left panels), after induction therapy (middle panels) and after dinutuximab beta therapy (right panels) for patients who achieved a complete response with induction therapy: Patient 1, Patient 4, Patient 6, and Patient 7. CT = computed tomography, MRI = magnetic resonance imaging.

**Figure 2:**
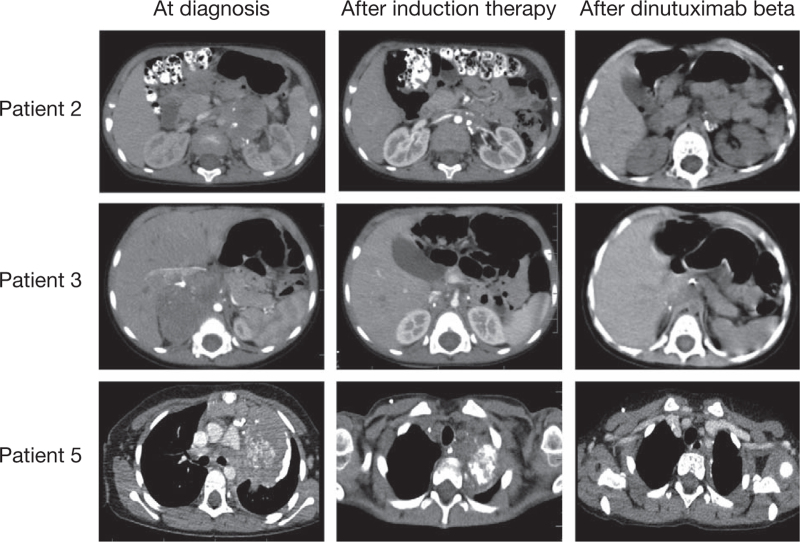
Abdominal/thorax CT scans at diagnosis (left panels), after induction therapy (middle panels) and after dinutuximab beta therapy (right panels) for patients who achieved a very good partial response with induction therapy: Patient 2, Patient 3, and Patient 5. CT = computed tomography.

All patients received multimodal therapy as described above. After induction therapy, 4 patients (Patients 1, 4, 6, and 7) were in complete remission (Fig. 1) and 3 (Patients 2, 3, and 5) achieved a very good partial response (VGPR, Fig. 2). All patients went on to receive consolidation therapy with BuMel and ASCT, with most patients undergoing partial or complete tumor resection either before or after consolidation therapy. Subsequently, the patients were treated with radiotherapy to the primary tumor and/or metastatic sites followed by immunotherapy with dinutuximab beta, consisting of 5 consecutive cycles. Immunotherapy was completed in 6 patients; in 1 patient (Patient 4), dinutuximab beta was permanently discontinued on the third day of cycle 1 due to severe neurotoxicity (cerebellar syndrome). High-dose intravenous immunoglobulins and corticosteroids were administered 48 hours after dinutuximab beta discontinuation and the symptoms of neurotoxicity completely resolved, with no pathology identifed on objective neurological examination.

Following completion of immunotherapy, the 4 patients who were in complete remission prior to dinutuximab beta (Patients 1, 4, 6, and 7) remained in complete remission, including the patient who interrupted dinutuximab beta due to toxicity (Fig. 1). The 3 patients with VGPR prior to immunotherapy (Patients 2, 3, and 5) achieved a partial response and received further treatment to eradicate residual disease: Patient 2 received 2 cycles of therapeutic MIBG with concomitant topotecan followed by a boost of peripheral blood stem cells, Patient 3 received a cycle of therapeutic MIBG with concomitant topotecan, and Patient 5 received proton-beam radiotherapy to the primary tumor site. After this additional treatment, Patients 3 and 5 achieved complete remission and Patient 2 demonstrated a VGPR. At the last follow-up in January 2022, 21 months after treatment completion, Patient 2 displayed stable disease, with no evidence of disease progression. The 6 patients in complete remission at the end of treatment remained disease-free, with a median duration of response of 21.5 months (range 9–30 months) as of January 2022.

AEs were largely manageable with supportive therapy and included mild-to-moderate pain, capillary leak syndrome, nausea/vomiting/anorexia, mild allergic reactions, irritability, hepatopathy, and hematological abnormalities (Table 2). Most AEs occurred during the first treatment cycle and decreased in frequency and intensity in subsequent cycles.

**Table 2:** Most common adverse events associated with dinutuximab beta therapy observed in our patient cohort.

Adverse event	Grading	Cycle 1 (7×)	Cycle 2 (6×)	Cycle 3–5 (18×)
Capillary leak syndrome	**II**	4	4	3
	**III**	3	–	–
Pain	**I + II**	1	2	3
Nausea/vomiting/anorexia/diarrhea	**I + II**	3	2	6
Ataxia/gait disturbance	**III**	1	–	–
Dysarthria	**II**	2	–	–
Allergic manifestations	**I + II**	3	2	5
Hepatopathia	**I + II**	6	5	12
Anemia	**I + II**	3	6	16
	**III**	4	–	–
Thrombocytopenia	**I + II**	1	3	3
	**III + IV**	3	1	–
Hypoalbuminemia	**I + II**	5	6	15
	**III**	2	–	–

## 4 Discussion

In this cohort of patients diagnosed with high-risk neuroblastoma in our clinic, dinutuximab beta was an effective maintenance therapy following induction and consolidation therapy, with 6 out of 7 patients achieving complete remission. Patients who demonstrated complete remission with induction therapy remained in complete remission after dinutuximab beta treatment and those who had a VGPR prior to dinutuximab beta treatment maintained their VGPR following immunotherapy completion. However, further treatment of those 3 patients with either therapeutic MIBG plus topotecan or proton-beam radiotherapy resulted in complete remission in 2 patients and VGPR in the other patient at end of treatment. As of January 2022, the treatment response is still ongoing, with a median duration of 21.5 months for the 6 patients who achieved complete remission. The patient with VGPR demonstrated stable disease 21 months after treatment completion.

Maintenance therapy with dinutuximab beta is the standard-of-care approach when treating high-risk neuroblastoma in Europe.^[9,12]^ Evidence from clinical studies with dinutuximab beta has demonstrated significantly improved survival for patients with high-risk neuroblastoma, with dosing strategies and toxicities well documented.^[4,8,9]^ Additional information from real-world studies and clinical cases is essential to help physicians to understand the optimal use of this immunotherapy in routine clinical practice.

In our patients, AEs were largely manageable with supportive therapy and included capillary leak syndrome, mild-to-moderate pain, and allergic reactions, in line with previously reported toxicities.^[4,8,9]^ In 1 patient, treatment was permanently discontinued due to severe neurotoxicity, which was completely reversible after appropriate therapy. In our clinic, we found that close patient monitoring and therapeutic measures, such as optimization of hydration, pain management, oxygen administration and antiallergic treatment could mitigate dinutuximab beta-associated AEs. Laboratory abnormalities noted with dinutuximab beta therapy were an increase of liver transaminase, hypoalbuminemia and a decrease in blood count parameters, consistent with other studies.^[4,8]^ As all patients underwent an intensive multimodal treatment regimen, these abnormalities were most likely influenced not only by dinutuximab beta itself but also by previous cytotoxic therapies. As per previously reported studies, both clinical adverse reactions and laboratory abnormalities during dinutuximab beta therapy were most frequent and intensive during the first cycle, with a decrease in both intensity and frequency of AEs in subsequent cycles.^[4,8]^ All dinutuximab beta-associated AEs observed in our clinic were reversible after treatment completion.

This case series has several important limitations to consider. As a result of its retrospective design, small sample size, short follow-up period and lack of comparison group, the value of the conclusions drawn from this study may be limited. Despite these limitations, our experience with dinutuximab beta as maintenance therapy confirms the benefits of this treatment in patients with high-risk neuroblastoma in routine clinical practice, complementing the findings of clinical trials. Given the risk of AEs associated with dinutuximab beta administration, the therapy should be provided at experienced centers of pediatric oncology whose educated staff is well trained to manage any potential complications of the therapy. Peer-to-peer experience of administering supportive therapies to manage AEs in real-world practice can provide critical evidence for using dinutuximab beta most effectively in the clinic.

## Acknowledgments

The authors thank Katrin Male of mXm Medical Communications for editorial support provided throughout the development of the manuscript.

## Author contributions

**Conceptualization:** Monika Achbergerová.

**Data curation:** Monika Achbergerová.

**Investigation:** Monika Achbergerová.

**Methodology:** Monika Achbergerová.

**Project administration:** Monika Achbergerová.

**Resources:** Monika Achbergerová.

**Supervision:** Monika Achbergerová.

**Writing – original draft:** Monika Achbergerová.

**Writing – review & editing:** Monika Achbergerová, Stanislava Hederová, Andrea Hrašková, Alexandra Kolenová.

## References

[1] Whittle SB Smith V Doherty E Zhao S McCarty S Zage PE. Overview and recent advances in the treatment of neuroblastoma. Expert Rev Anticancer Ther 2017;17:369–86. 28142287 10.1080/14737140.2017.1285230

[2] Cohn SL Pearson AD London WB. The International Neuroblastoma Risk Group (INRG) classification system: an INRG Task Force report. J Clin Oncol 2009;27:289–97. 19047291 10.1200/JCO.2008.16.6785 PMC2650388

[3] Park JA Cheung NV. GD2 or HER2 targeting T cell engaging bispecific antibodies to treat osteosarcoma. J Hematol Oncol 2020;13:172. 33303017 10.1186/s13045-020-01012-y PMC7731630

[4] Ladenstein R Pötschger U Valteau-Couanet D. Interleukin 2 with anti-GD2 antibody ch14.18/CHO (dinutuximab beta) in patients with high-risk neuroblastoma (HR-NBL1/SIOPEN): a multicentre, randomised, phase 3 trial. Lancet Oncol 2018;19:1617–29. 30442501 10.1016/S1470-2045(18)30578-3

[5] Nazha B Inal C Owonikoko TK. Disialoganglioside GD2 expression in solid tumors and role as a target for cancer therapy. Front Oncol 2020;10:1000. 32733795 10.3389/fonc.2020.01000 PMC7358363

[6] Morandi F Sabatini F Podestà M Airoldi I. Immunotherapeutic strategies for neuroblastoma: present, past and future. Vaccines (Basel) 2021;9:43. 33450862 10.3390/vaccines9010043 PMC7828327

[7] Perez Horta Z Goldberg JL Sondel PM. Anti-GD2 mAbs and next-generation mAb-based agents for cancer therapy. Immunotherapy 2016;8:1097–117. 27485082 10.2217/imt-2016-0021 PMC5619016

[8] Ladenstein R Pötschger U Valteau-Couanet D. Investigation of the role of dinutuximab beta-based immunotherapy in the SIOPEN high-risk neuroblastoma 1 Trial (HR-NBL1). Cancers (Basel) 2020;12:309. 10.3390/cancers12020309 PMC7072500 32013055

[9] EMA . Qarziba (Dinutuximab beta) Summary of Product Characteristics. Available at: https://www.medicines.org.uk/emc/product/9441/smpc#gref. Accessed April 2021.

[10] Keyel ME Reynolds CP. Spotlight on dinutuximab in the treatment of high-risk neuroblastoma: development and place in therapy. Biologics 2019;13:01–12. 10.2147/BTT.S114530 PMC6306059 30613134

[11] Achbergerová M Hederová S Mikesková M Husáková K Hrašková A Kolenová A. Implementation of immunotherapy into the treatment of neuroblastoma - single center experience with the administration of dinutuximab and management of its adverse effects. Klin Onkol 2020;33:372–8. 33108882 10.14735/amko2020372

[12] Smith V Foster J. High-Risk neuroblastoma treatment review. Children (Basel) 2018;5:114. 10.3390/children5090114 PMC6162495 30154341

